# Selection Affects Genes Involved in Replication during Long-Term Evolution in Experimental Populations of the Bacteriophage φX174

**DOI:** 10.1371/journal.pone.0060401

**Published:** 2013-03-22

**Authors:** Celeste J. Brown, Jack Millstein, Christopher J. Williams, Holly A. Wichman

**Affiliations:** 1 Department of Biological Sciences, University of Idaho, Moscow, Idaho, United States of America; 2 Institute for Bioinformatics and Evolutionary Studies, University of Idaho, Moscow, Idaho, United States of America; 3 Department of Statistical Science, University of Idaho, Moscow, Idaho, United States of America; University of Massachusetts Medical School, United States of America

## Abstract

Observing organisms that evolve in response to strong selection over very short time scales allows the determination of the molecular mechanisms underlying adaptation. Although dissecting these molecular mechanisms is expensive and time-consuming, general patterns can be detected from repeated experiments, illuminating the biological processes involved in evolutionary adaptation. The bacteriophage φX174 was grown for 50 days in replicate chemostats under two culture conditions: *Escherichia coli* C as host growing at 37°C and *Salmonella typhimurium* as host growing at 43.5°C. After 50 days, greater than 20 substitutions per chemostat had risen to detectable levels. Of the 97 substitutions, four occurred in all four chemostats, five arose in both culture conditions, eight arose in only the high temperature *S. typhimurium* chemostats, and seven arose only in the *E. coli* chemostats. The remaining substitutions were detected only in a single chemostat, however, almost half of these have been seen in other similar experiments. Our findings support previous studies that host recognition and capsid stability are two biological processes that are modified during adaptation to novel hosts and high temperature. Based upon the substitutions shared across both environments, it is apparent that genome replication and packaging are also affected during adaptation to the chemostat environment, rather than to temperature or host per se. This environment is characterized by a large number of phage and very few hosts, leading to competition among phage within the host. We conclude from these results that adaptation to a high density environment selects for changes in genome replication at both protein and DNA sequence levels.

## Introduction

Determining the biological processes that are affected when organisms adapt to their environment is an important step in understanding the evolutionary process. The molecular changes that arise in DNA or protein sequences during adaptation affect phenotypic traits and fitness via these biological processes. Following the connections from genome sequence to biological processes and fitness in complex organisms is difficult and often relies upon finding a preponderance of adaptive substitutions in genes affecting the same biological process [1]–[3]. Another way to make connections between sequence, process and fitness is by experimental evolution [4]. For example, experimental adaptations of *Escherichia coli* have related increased fitness to global changes in genome sequence and transcriptional networks [5]–[7]. However, even the *E. coli* genome is so large and complex that the results of fine-scale dissection of the biological processes involved in adaptation are often ambiguous unless the selection is very specific [8], [9].

Bacteriophages and their hosts are a common model for experimental evolution studies [10]–[18]. The size of phage genomes, ease of culturing and rapid generation time make them ideal for studying the molecular basis of adaptation in a selective environment. The small microvirid phage, φX174, is particularly suitable because its genome is exceptionally small, its genome structure is simple, and its molecular and structural biology are well-characterized [19]–[22]. Table 1 shows the gene names and protein functions for each of the 11 genes of the φX174 genome, as well as the biological processes in which they participate. Almost all molecular functions related to replication, transcription and translation are performed by the host cellular machinery. In environments in which host evolution is minimized, adaptive changes accumulate mainly in the phage genome [23]. Thus, bacteriophage grown under such conditions provide an experimental system in which biological processes involved in adaptation can be studied easily.

**Table 1:** Biological processes in which φX174 proteins participate.

Gene	Protein Function	Biological Process	Obs/Exp †
*A*	Replication Initiation	Replication, DNA packaging	44/29
A*	Endonuclease	Unknown	16/19
*B*	Internal Scaffold	Capsid assembly	2/7
*C*	DNA/Protein Binding	DNA packaging	3/5
*D*	External Scaffold	Capsid assembly	2/9
*E*	Lysis	Host-phage interaction	3/5
*F*	Major Capsid	Host recognition, Capsid assembly, Capsid structure	24/24
*G*	Major Spike	Host recognition, Capsid assembly, Capsid structure	1/10
*H*	Pilot or Minor Spike	Host recognition, Capsid assembly, Capsid structure, DNA transport, Replication	16/18
*J*	ssDNA Binding	DNA packaging, Capsid assembly, Capsid structure	1/2
*K*	Non-essential	Unknown	2/3

† Percent of sites with observed (Obs) substitutions in each gene compared with the percent expected (Exp) due to the length of the gene. A* is a subset of A. Values do not add to 100% due to overlapping genes.

Previous evolution experiments with φX174 illustrate many consequences of short-term selection under varied environmental conditions [14], [15], [17], [24]–[27]. Some of these experiments used a two-stage chemostat to minimize adaptation in the host. A two-stage chemostat grows the host cells in the first stage, and then transfers growing cells at a constant rate into the second stage in which there are phage. The results of these studies have shown consistently that phage adapt to new hosts, new temperatures or the high multiplicity of infection environment of a chemostat through the accumulation of many common substitutions and several unique substitutions [28]. The common substitutions reveal much about the biological processes involved in adaptation. For example, during growth on *Salmonella typhimurium*, adaptive changes occur in the major capsid protein, which directly interacts with components of the bacterial cell wall. These adaptations can be reversed when the phage are grown on *E. coli* [25]. There are also adaptive changes that occur in the major capsid protein during growth at high temperature [29]. These results suggest that host recognition and capsid assembly are important biological processes involved in adaptation to these environmental challenges.

For the data presented here, replicate two-stage chemostats for each of two environments, 37° with *E. coli* C as the host and 43.5° with *S. typhimurium* LT2 as the host, were grown for 50 days. These two environments were meant to bestow weak and strong selection, respectively. Over 50 years ago φX174 was domesticated by growing on *E. coli* at 37° [30] and doubles its population size 20 times per hour when grown in batch culture under these conditions [31]. Hence this combination of host and temperature should be benign and selection due to these conditions should be weak. On the other hand, *S. typhimurium* at 43.5° is both a novel host and a non-permissive temperature for the lab-adapted strain of φX174, whose population only doubles nine times per hour under these conditions [31]. Hence selection in this environment is quite strong [24], [26]. Additionally, the experiments presented here were continued over a longer time period than most previous experiments, which lasted 10 or 11 days up to 22 days, and minimally covered 3600 phage generations [27], [28]. The longer time period was specifically chosen to observe whether selection persisted within the chemostat environment once the initial adaptation to host and temperature was attained. The rate of molecular evolution was compared among the chemostats presented here, and the comparison indicated that, although initially the strong selection environment had a greater evolutionary rate, both environments continued to evolve at approximately the same rate for the full 50 day period [23]. Indeed, one of the chemostats growing on *E. coli* at 37° was continued for another 130 days, and adaptation to this environment persisted over the entire time [27]. The persistence of evolution over time was attributed to competition for host cells in the chemostat environment, where phage greatly outnumber the available hosts [23].

Here we present the complete sequence data for these four chemostats and compare them to genetic variation found in wild phage. Counts of sequence changes in these chemostats were previously summarized in [23] and [28], and an analysis of the 180 day chemostat was presented in [27]. We compare the results from these long term chemostats to previously described experiments at a level of detail that allows us to provide new insights into the biological processes that may be affected by these adaptive changes and the specific selection pressures, temperature, host or high multiplicity of infection, driving evolution in these experiments.

## Materials and Methods

### Strains

A lab-adapted strain of φX174 was used as the ancestor for these studies (GenBank Accession: AF176034). The bacteria used for phage propagation were *Escherichia coli* C and the Type I restrictionless (*hsd)*, CpX 174^s^ *Salmonella typhimurium* LT2 strain IJ750 [*xyl-404 met A 2 2 metE551 galE7I 9 trpD2 ilv-452 hsdLT6 hsdSA29 hsdSB121 fla-66 rpsLI20* H1-b H2-e *nix].* These are the same phage and bacterial hosts used in previous similar experiments [24], [25].

### Two-stage chemostat

A chemostat consisting of two 100×15 mm glass test tubes was used to select phage populations as previously described [24] except that the media (LB broth: 10 g NaCl, 10 g Bacto Tryptone, and 5 g yeast extract/liter, 0.1% antifoam B, Sigma A5757) contained 2 mM CaCl_2_. Briefly, host cells were maintained at a density suitable for phage growth in the first tube, and media containing cells from this tube was drawn continuously into the second tube, which contained phage. The volume of the phage tube was replaced on the order of 100 times per day, which is equivalent to a growth of 144 phage population doublings per day given the continuous nature of replacement [27]. The chemostat was reassembled every two days and inoculated with 0.2 mL of filtered phage lysate, thus discarding any phage-resistant cells.

### Sequencing

Whole genome sequencing was carried out as previously described [27]. In most cases, the sequence was determined from a PCR of the heterogeneous lysate from the chemostat culture (referred to as “population consensus” sequences). Data for *E. coli* C, 37° chemostats came from both a population consensus and zero to four isolates. One of the *E. coli* C, 37° chemostats evolved an interfering particle [23], and sequence changes were determined only from two isolates at each time point while the particle was present. These data are reported simply as changes that were detected at a particular time point, and combining the isolate data this way makes them roughly equivalent to the population-derived consensus sequence. A consensus sequence does not identify changes present at low or moderate frequency in the culture and can exhibit genuinely ambiguous bases where polymorphisms occur at intermediate frequency, but it also provides an expedient means of identifying changes that are common in the population.

### Statistical analysis

Substitutions occurring in the four 50-day chemostats were compared to those observed in previous evolution experiments. Chemostat experiments previously described in Wichman and Brown [28] can be divided into types based upon temperature and host (Table 2). These experiments were conducted in chemostats that lasted from 10 to 22 days under four conditions: the host was *S. typhimurium* and, the temperature was 42° to 43.5° (S42); the host was *E. coli,* and the temperature was 37° (E37); the host was *E*. *coli,* and the temperature was 42° to 43.5° (E42); and the host was *S. typhimurium,* and the temperature was 37° (S37). Hypotheses developed based upon the chemostats in the present study, i.e., the index chemostats grown on *E. coli* at 37° or *S. typhimurium* at 43.5°, were tested using the data from these other chemostats, i.e. the test chemostats, thus providing an independent test of our hypotheses (Table 2). The hypotheses of interest were whether or not a set of substitutions occurred more frequently under certain chemostat conditions. The sets of substitutions were determined by their distribution among the index chemostats: found in both chemostat environments, in only the *S. typhimurium* at 43.5° chemostats or in only the *E. coli* at 37° chemostats. Thus the average number of substitutions from each set that were found in each experiment were compared among chemostat conditions. Since the distributions of the numbers of substitutions were nonnormal, Kruskal-Wallis (KW) tests were used to test each hypothesis and permutation-based P-values were computed [32]. When follow-up comparisons between pairs of conditions were performed, permutation-based Wilcoxon tests were used [32]. To test the hypotheses of 1) proportional distribution of substitutions among genes, or 2) homogeneity of gene substitution proportions between experimental and wild phage, exact chi-squared tests were conducted [32]. Other hypotheses concerned the distribution of individual substitutions across experimental conditions and were tested using Fisher's exact test [32]. For situations in which a difference was found between conditions in the index experiments, we might confirm this difference in the test experiments. However, for situations where there were no differences between conditions in the index experiments, if we fail to detect a difference in the test experiments then we typically will not have enough power to definitively conclude that no difference exists. We can, however, note when the test experiment result is consistent with the index experiment result.

**Table 2:** Number of chemostat experiments for each environmental condition in the index and test sets and the publications in which sequence information was presented.

Set	Reference	Environment*			
		S42	E37	E42	S37
Index	This study, [27]	2	2	0	0
Test	[24]	4	0	5	0
	[26]	2	0	0	0
	[17]	0	0	1	0
	[25]	3	0	2	0
	GenBank AF299300 – AF299314	5	5	0	1
	Unpublished data	1	3	0	2

* S = *S. typhimurium*, E = *E. coli*, 42≥42°, 37 = 37°.

## Results and Discussion

Four experimental lineages were started from independent isolates of the same ancestral strain of φX174 and grown in two-stage chemostats for 50 days under constant conditions: two lineages were grown on *Escherichia coli* C at 37° (designated E37a and E37b), and two on *Salmonella typhimurium* LT2 at 43.5° (S43a and S43b). The populations of these phage lineages progressively accumulated changes throughout their 50-day history, assayed every 10 days. Frequencies of some substitutions that arose in these chemostats were previously summarized in [23] and [28]. E37b developed an interfering particle as reported in [23]. E37a was run for a total of 180 days, and some of the sequence data for E37a were presented previously [27]. We first provide a summary of the genetic changes that arose in each chemostat, and then we discuss the biological processes that might have been affected by these changes.

**Figure 1:**
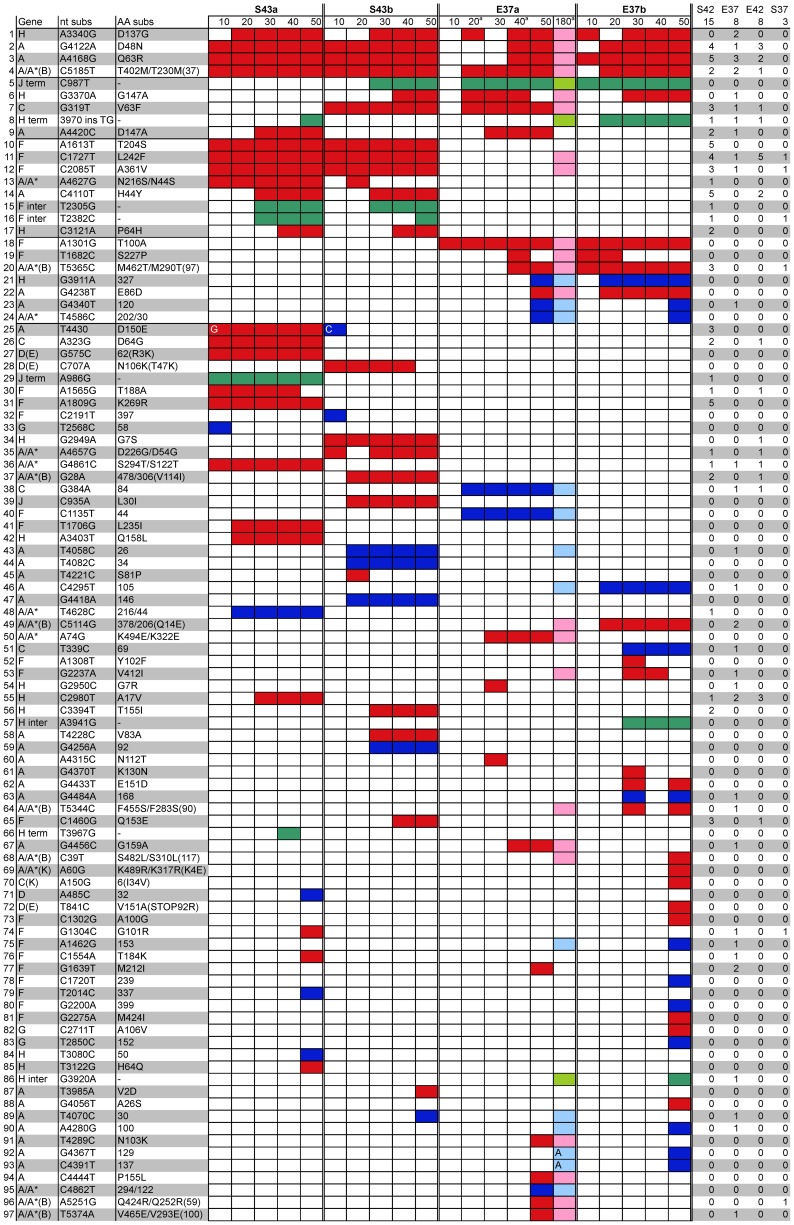
Substitutions detected in four chemostats over 50 days. S43a and S43b were grown on *S. typhimurium* as host at 43.5°; E37a and E37b were grown on *E. coli* at 37°. Order by row is based upon the number of chemostats in which the substitution arose, then on the earliest 10 day sample in which it was detected and then by position in the genome. The column labeled ‘180’ indicates the presence of a substitution in E37a after 180 days of evolution [27]. The last four columns indicate the number of other chemostat experiments ([17], [24]–[26], unpublished data) in which the substitution arose at each environmental condition (S = *S. typhimurium*, E = *E. coli*, 42≥42°, 37 = 37°). Red indicates missense substitutions, blue silent substitutions and green substitutions in intergenic regions. ^a^Previously presented in [27].

The substitutions that arose to detectable levels in the four 50-day chemostats are presented in Figure 1. (For the reader's convenience, data reported in [27] are also presented in columns marked by a superscript a.) Missense substitutions are indicated by red, silent substitutions by blue and intergenic substitutions by green. To highlight parallel changes that occurred in this study, the order of presentation is based upon number of chemostats in which the substitution arose, then on the earliest sample in which it was detected, and then by position in the genome based upon the sequence. Thus, rows 1–9 are substitutions that arose in both environments, rows 10–17 arose in both S43 chemostats, rows 18–24 arose in both E37 chemostats, and rows 25–97 arose only in a single chemostat in these experiments. For easy comparison to other similar experiments, the far right columns indicate the number of times that a substitution has been detected in a particular chemostat environment in other similar experiments. These are the test set in Table 2. Below the labels designating environment are the number of other experiments that were conducted under the listed conditions. So, for instance, S42 indicates chemostats at 42° or higher with *S. typhimurium* as host, and the number of other experiments with these conditions was 15.

In the 50-day chemostats, the substitutions that were found in both environments were in genes involved in genome replication and packaging – the replication initiation protein A (4), the pilot protein H (2), the DNA maturation protein C (1) – and in two intergenic regions (Figure 1, rows 1–9). Most of these substitutions have been seen in other experiments including ones that arose under a different environmental condition (*E. coli* host at >42°). The wide distribution of these substitutions under different environmental conditions led us to test the hypothesis that the average number of these substitutions per experiment are equal among environments. Using only the data from previous experiments (test set) and excluding the information from the 50-day chemostats, we failed to reject this hypothesis (KW  = 2.55, df  = 3, P = .51). As mentioned above, we do not have enough power to conclude that no difference exists, but these results are consistent in direction with the index experiment results. The similar distribution of these substitutions across multiple experimental conditions in both the index set and the test set suggests that a feature that is common to all environments, such as the high multiplicity of infection that exists within the chemostats [23], [27], was the selective pressure to which these populations were responding.

The substitutions that arose in both S43 50-day chemostats are in the major capsid protein F, the replication initiation protein A, the pilot protein H, and the intergenic region between *F* and *G* (Figure 1, rows 10–17). All of these substitutions have been seen in other experiments, but predominantly in *S. typhimurium* at 43°. The test of equality of the average number of these eight substitutions among the four environments in the test experiments was rejected (KW  = 9.75, df  = 3, P = .014), supporting the alternative hypothesis that these substitutions are specifically adaptive in the S43 environment. We infer, as have others, that these substitutions are responding to either the novel host, the high temperature or both [17], [29].

Substitutions that were detected in both E37 50-day chemostats were also in the F and A proteins, and three substitutions were silent in A or H (Figure 1, rows 18–24). Only one of these, T5365C, has been seen in more than one other experiment. Using the test set, the test for homogeneity of proportion across the four conditions for this single site is not significant (Fisher's exact *χ* ^2^  = 4.5, df  = 3, p = 0.20), a result which is NOT consistent with the index experiments. The three parallel silent substitutions are intriguing. Although silent sites are generally considered to evolve neutrally, there are several hypotheses for why this may not necessarily be true. These sites may be important in the folding of the ssDNA genome within the capsid or during ejection. The relationship between silent substitutions and codon bias in gene A of φX174 has been discussed previously [27]. We present a further possibility below. Each of these hypotheses require further investigation.

A large fraction of substitutions arose in only a single 50-day chemostat (Figure 1, rows 25–97). These unique substitutions include all of those that arose in genes *D* or *E, J, G,* and *K*, as well as almost half (20 of 43) of the substitutions that arose in gene *A*. Of the 73 substitutions that were seen in only one chemostat in this study, 35 were found in previous studies including eight that arose in E37a after day 50. Note, however, that the experiments presented here are the first that were run for more than 22 days, and the vast majority of earlier experiments were run for only 10 or 11 days. Almost all of the truly unique substitutions were detected after day 10, so it is possible that the lack of parallel evolution at some of these sites is an artifact of the shorter length of these earlier experiments.

### 

#### Substitutions in sequences involved in transcription

There are three promoters and four transcription terminators in φX174. In the 50-day chemostats, substitutions arose in the −35 sigma factor binding site of one of the promoters: G319T, which was seen in S43b and E37a (Fig. 1, row 7), and A323G, which was seen in S43a (Fig. 1, row 26). These substitutions are functionally equivalent in that they down-regulate transcription from this promoter and are adaptive at high temperature [31]. These substitutions also affect the amino acid sequence of protein C (see below), however, adaptive substitutions at adjacent silent sites have similar effects on transcription and a slightly smaller effect on fitness.

There are two adjacent substitutions immediately downstream of the first transcription termination signal, A986G, which arose in S43a (Fig. 1, row 29), and C987T, which arose in the other three 50-day chemostats (Fig. 1, row 5). Predictions of the strength of this rho-independent transcription terminator are unaffected by these substitutions, however, the C987T substitution increases the number of Ts in the T-tail, which affects readthrough of the RNA polymerase to the downstream genes [33], [34]. This site is also an extra-origin cleavage site for the endolytic activity of A/A* and both of these substitutions alter conserved nucleotides in this site [33], [35]. Determining whether these intergenic substitutions are functionally equivalent and whether they affect mRNA or genome stability awaits further experimentation.

#### Substitutions in genes involved in phage genome replication and packaging

**Figure 2:**
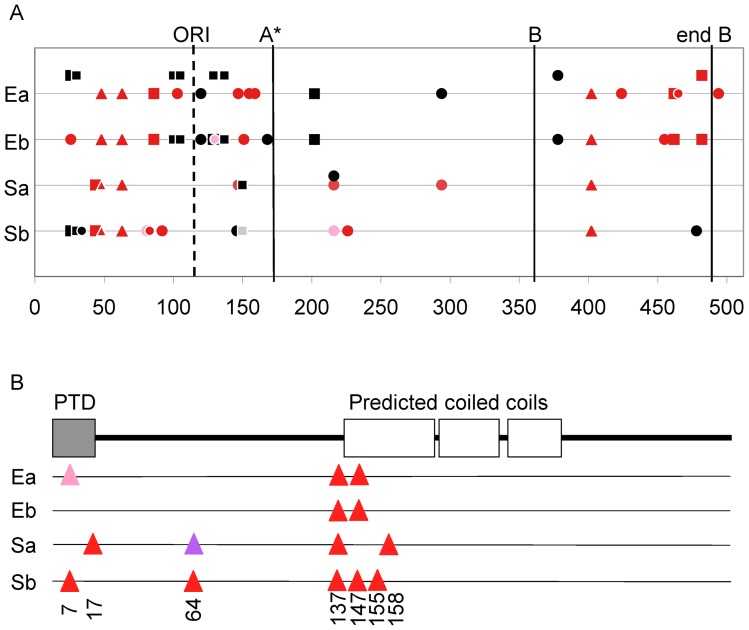
Distribution of substitutions in proteins A and H are not uniform. A) Substitutions in the 512 codons of gene *A*. The dashed vertical line indicates the origin of replication, the other vertical lines indicate the starts of A* and B, and the end of B (residues: A = 1–512; A* = 173–512; B = 364–484). Red indicates missense substitutions and black indicates silent substitutions in genes *A* or A*, not *B*. Fainter colors indicate the substitution was not detected at day 50. Triangles mark substitutions found in all four chemostats, squares mark substitutions found in more than one chemostat and circles are unique to each chemostat. Sa = S43a, Sb = S43b, Ea = E37a, Eb = E37b. The symbols above the line for Ea indicate parallel substitutions that arose in Ea by 180 days [27]. The black circle above the Sa line indicates a silent substitution that affected the same amino acid as the red circle on the line. B) Substitutions in protein H. Graphic of predicted domains from [64]. Red triangles indicate missense substitutions, pink indicates the substitution was not detected at day 50, and purple indicates a second missense substitution at that residue. The vertical numbers indicate position in the protein sequence. PTD = predicted transmembrane domain. Not to scale.

The proteins involved in genome replication and packaging are A, H and C. The replication initiation protein A accrues the most substitutions in these experiments. Fully 44% of all substitutions in the 50-day chemostats were in the A gene, and two thirds of these were in the N-terminal third of the protein (Fig. 2A, Table 1). The distribution of substitutions among genes is significantly different than expected given their lengths in nucleotides (Fisher's exact χ^ 2^  = 21.6, df  = 7, P = 0.005). The greatest contribution to this χ^2^ value is from the excess number of substitutions in gene A (χ^2^  = 7.9, df  = 1, P<0.005).

Gene *A* contains the coding sequences for proteins A, A* and B and the N-terminus of protein K, as well as the origin of replication (*ori*). Protein A* shares all but the first 172 residues with protein A and also shares many of the functions of A except the ability to initiate rolling circle replication and package viral DNA into the capsid [36]. The N-terminal third of A interacts with the host helicase, which unwinds the DNA during viral strand synthesis [37], [38], and probably with host single-stranded DNA binding protein [35]. The replication initiation protein A acts in *cis* to initiate rolling circle replication, which is accomplished by concomitant transcription, translation and binding to the dsDNA from which transcription was initiated [39], [40]. Because protein A only aids in the replication of its own DNA, directional selection may act on this protein to increase replication without danger of being hijacked by coinfecting phage, and this may account for the large number of substitutions in A relative to the other genes.

Three substitutions in protein A have been found in 12 (G4122A), 14 (A4168G) and 9 (C5185T) experiments (Fig. 1, rows 2–4). These substitutions are also found in or, in the case of 5185T, are fixed in wild φX-like phage [28]. The proportion of each of these substitutions is not significantly different among the various environments (S42, E37, E42, S37) when the test set is used (Fisher's exact test, P-values: 0.4–0.95), a result which is consistent in direction with the index experiments. One constant among these environments is their high MOI, a condition that may well occur in the waste water treatment plants from which the wild phage were collected. Thus, competition with conspecifics may select for adaptation in the process of genome replication. The elucidation of the exact mechanism by which these adaptive substitutions affect replication awaits further study.

An advantage of the nucleotide substitutions in gene A, both silent and missense, may take the form of improved DNA binding affinity for the helicase or faster DNA unwinding, such that new viral strands are produced more quickly [41]. The accumulation of substitutions around the *ori* in E37b may allow faster DNA unwinding (Fig. 2A). Of the 12 substitutions in this region, both missense and silent, seven were G to T transversions and two were C to T transitions. Overall this led to a decrease in the GC content around the *ori* and especially downstream. A similar clustering of substitutions in this region occurred in the E37a chemostat after day 50 [27]. In this case, the predominance of silent substitutions led to a pronounced increase in GC content upstream of the *ori*. Of the 17 sites upstream of the *ori* that underwent substitutions, five were G or C and 12 were A or T in the ancestral phage. By day 180, 13 of these sites were G or C and only four were A or T. This was primarily due to transitions from A or T to G or C.

Both of these results are consistent with the mechanism of rolling circle replication and suggests a selective advantage for the silent as well as missense substitutions in gene A. A reduction in GC content in the direction in which the helicase unwinds the dsDNA would potentially reduce the energy cost and increase the speed of unwinding. On the other hand, the DNA replication machinery requires a double-stranded template from which to start replication. Increasing the GC content upstream of the *ori* may strengthen the double-stranded nature of the template DNA. That these increases in silent substitutions occur very late suggests that they are mutations of small benefit with relatively weak selective advantage and can only accrue when the population size is quite large and when they are not competing with mutations of larger effect. This could explain the dearth of such mutations in S43a (Figure 2A), where the titer was significantly lower than the other chemostats by day 50 [23].

Protein H is a multi-functional protein that has been associated with many biological processes, including ejection and replication (Table 1). Protein H, also called the pilot protein, brings the viral strand into the cell (ejection) and transports it to sites for DNA replication of ssDNA to dsDNA [42]–[44]. It has been proposed that the N-terminal transmembrane helix mediates the transition across the bacterial cell wall [45]. Thus, substitutions in this region, especially C2980T (Fig. 1, row 55), which has been found in seven experiments, may affect ejection. The number of sites for second-strand synthesis are limited [46], and other substitutions in protein H may be due to competition for the host replication machinery. The locations of multiple and parallel substitutions in the 35 amino acids from 136 to 170 suggest that this region, immediately adjacent to and within the first coiled coil domain, is important in the chemostat environment. Over multiple evolution experiments there have been 31 substitutions at sites including five sites that have undergone parallel substitutions in multiple environments [28]; and unpublished data. These environments include three bacterial host species (*E. coli, S. typhimurium, Shigella sonnei*) and are evenly divided between chemostats run at either 37°C or >42°C. Removing the index set (17 sites), the test for homogeneity of proportions across environments is not significant (Fisher's exact χ^2^  = 6.4, df  = 3, P = 0.1), a result which is consistent in direction with the index experiments.

Two non-coding regions appear to be important for replication from ssDNA to dsDNA. One is in the intergenic region between genes H and A. This sequence may interact with a component of the host replication machinery that is present in limiting amounts, possibly the sites for second-strand synthesis mentioned above [47], [48]. Competition for this host factor may drive selection in this intergenic region. There are 4 substitutions in this region, one that was only present at detectable levels on day 40 (Fig. 1, line 66), and one that occurred in several experiments under a number of environmental conditions (Fig. 1, line 8). This number of substitutions, let alone parallel substitutions, is much greater than would be expected by chance given the short length (64 nt) of this region. However, these two substitutions are also in the predicted transcription termination signal for H. Determining whether they affect replication or transcription awaits further experimentation. The other non-coding region that is important for ssDNA to dsDNA replication is the intergenic region between F and G (F inter). One substitution that arose in this region in the S43 chemostats and in a third experiment (T2305G; Fig. 1, row 15) is within the primosome binding site [49], and substitutions in this site may improve its interaction with the *S. typhimurium* primosome.

Protein C is involved in packaging ssDNA into the capsid (Table 1). This protein must bind to the dsDNA prior to binding by host replication factors in order to start packaging ssDNA into the procapsid. As well as down-regulating transcription from the D promoter, substitutions at G319T and A323G alter adjacent amino acids in protein C, V63F and D64G, respectively [50]. These two sites have arisen in 11 experiments, but never in the same phage. In addition, during host switching experiments in which a number of substitutions in the capsid proteins reverted to the original amino acid, V63F also reverted, suggesting that it may be interacting with either the capsid or the host helicase. The number of times that these substitutions arose in chemostats that had *E. coli* C as the host (4), however, suggests that it is the capsid with which they are interacting.

These substitutions also affect transcription, as mentioned above, and they may also be affecting genome replication. The transcription and replication machinery move in the same direction around the phage genome. Studies in *Bacillus subtilis* show that these two machineries may interfere with each other [51]. Down regulating expression from the D promoter may decrease the amount of transcript for the capsid proteins, however, the B promoter still produces transcripts that include these proteins. It is an intriguing possibility that the loss in transcript due to down regulating the D promoter results in a sufficiently faster replication rate that increases fitness of phage carrying these regulatory substitutions.

#### Substitutions in genes involved in host recognition

Another process in which protein H is involved is binding to the cell receptor [42], [43], [52]. The substitutions at amino acid 64 that have only been found in S43-like chemostats may well be involved in binding to the cell receptor, which differs in structure between *E. coli* and *S. typhymurium* [53].

Protein F, the major capsid protein, is involved in recognizing and attaching to the host lipopolysaccharide. The substitution in F that persisted throughout the duration of the two E37 chemostats, A1301G, alters the amino acid sequence at a site that is adjacent to two sites that are known to be important for host recognition [25], [54]. The three substitutions in F that arose in both S43 chemostats (Fig. 1, rows 10–12) have arisen in multiple experiments and have been attributed to adaptations to either novel host (C2085T), high temperature (C1727T) or both (A1613T) [17], [29]. Thus, some of the substitutions in F can be attributed to its role in host recognition and attachment and some to capsid assembly. Interestingly, two of these three substitutions in F eventually arose in E37a after this chemostat was continued for another 130 days (Fig. 1, rows 11,12). It seems likely that these substitutions have a smaller but positive selective advantage in that environment as well.

#### Substitutions in genes involved in capsid assembly

The major capsid protein F interacts with proteins B, D, G, J and H during capsid assembly and is part of the capsid [19], [55]. The role of adaptive substitutions in protein F has been discussed extensively and recently reviewed in [28], therefore we will not discuss them here. It is interesting to note, however, that there were very few substitutions in genes *B, D, G* and *J*. The possibility that substitutions in F are interacting with substitutions in H has not yet been explored.

#### Distribution of substitutions compared to wild phage

The large number of substitutions in gene *A* could indicate simply that *A* is hypermutable, or the fitness effect of changes in these genes is nearly neutral. Similarly, the dearth of substitutions in genes *D* or *E, J, G,* and *K* may be because they are highly conserved genes. If these explanations hold then the amount of variation in natural populations should reflect the variation seen in the chemostats. We compared our results to 16 natural microvirid phage isolates in the φX174 clade [56]. In these 16 isolates, there are substitutions at 706 sites relative to the wild phage consensus sequence; 144 (20%) are missense, 545 (77%) are silent and 18 (3%) are intergenic. Substitutions shared among wild phages are assumed to be identical by descent, although this is a simplifying assumption. The distribution of substitutions among genes is significantly different between the 50-day chemostats and the wild phages (*χ* ^2^ = 35.3, df = 6, P<0.001).

**Figure 3:**
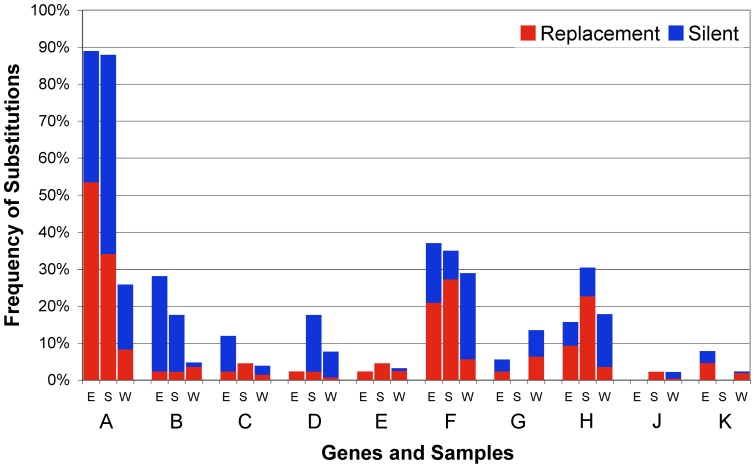
Distribution of substitutions is different between chemostats and wild phage. Percent of all missense (red) and silent (blue) substitutions for each environment (E =  E37, S = S43) and among wild phage (W) [56] by gene (A–K).

Figure 3 shows the frequency of sites with missense and silent substitutions for each gene in the wild phages and in each environment. A* is not included as it is an in-frame variant of A. For seven of the remaining 10 genes, the wild phages have the typical pattern of substitutions for genes undergoing purifying selection; there are far fewer missense substitutions (20%) than would be expected under a neutral model of evolution, which for this genome is about 77% of sites. The remaining three genes overlap with the coding sequence of other genes, *B* with A/A*, E with *D*, and *K* with A/A* and *C*. In the wild phage, about 75% of the substitutions in *B, E* and *K* are missense substitutions, and these are generally silent in their overlapping genes. The apparent neutral evolution of these three genes has been noted previously [56]. Thus, the frequency of substitutions in the chemostat experiments does not reflect the frequency of substitutions in natural populations of phages, and we can reject the notion that the patterns seen in the chemostat experiments reflect the degree of conservation of the individual genes.

Although the overall distribution of missense and silent substitutions suggests that purifying selection is the predominant force in natural populations of phage, there are indications of similar adaptive mechanisms in natural and experimental phage populations [28]. Twenty seven of the substitutions in the 50-day chemostats are also found in the wild phage populations, and this is twice what would be expected by chance (13). In four cases, the adaptive substitution is a reversion to the monomorphic wild phage nucleotide: C5185T and A3340G occurred in all four chemostats; G384A, which is a silent substitution in gene C, arose and persisted in only a single chemostat; C1727T arose in the S43 chemostats and eventually in E37a. This last substitution is a well known response to growth at high temperatures that produces a leucine to phenylalanine substitution in the capsid protein F [29]. Its presence in wild populations of phage suggests that they experience high temperatures in nature. If C5185T and A3340G are indeed arising in response to high MOI, then such conditions may also exist in nature.

At other sites, the wild phage are polymorphic at the same sites that mutated in the 50 day chemostats. At three sites (T4628C, C1460G, C2191T), the ancestral sequence is the same as the less common sequence in the wild phage, and adaptive substitutions are convergent to the consensus sequence for the wild phage. At 18 sites (G4256A, T4289C, C4391T, A1809G, G1304C, A4168G, C2085T, T4058C, C39T, G4122A, C4110T, A986G, T4082C, G4418A, C2980T, C1720T, T2014C, G2275A), the ancestral sequence is the same as the wild phage consensus, and the substitution is to a nucleotide that is found in at least one wild phage. Two sites (G4367T, C2711T) had two low frequency substitutions in the wild phage: one is the same as the experimental substitution and one is different. Four sites (G575C, T3122G, A1308T, G4238T), are polymorphic in the wild phage, but did not have the same substitution as the experimental phage. The remaining 66 sites are monomorphic in the wild phage. Thus about a third of the adaptive substitutions that were seen in these experiments are also found in natural populations of phage, suggesting that evolutionary pressures found in these experiments are also found in nature [28].

## Conclusions

This study provides valuable insights into the biological processes that are affected when φX174 adapts to a chemostat environment. Although the primary force leading to adaptation in the S43 chemostats appears to be related to host recognition or temperature, the secondary force in these chemostats and the primary force in the E37 chemostats appears to be competition amongst the phage. Two steps are obvious from these and other experiments [26], [27]. In chemostats with novel hosts or temperatures, substitutions arise first in genes involved in host recognition and capsid assembly. Thus the greatest initial increase in fitness comes from these processes, leading to increases in population numbers within the chemostat [23]. The second evolutionary step, therefore, is adapting to high viral density where there is intense within-host competition. One way to outcompete coinfecting conspecifics is to have a higher growth rate. Consistent with this, the genes that appear to have accumulated substitutions in both environments are involved in genome replication and packaging.

Genome replication is an obvious target of selection for higher growth rate. Indeed, drugs that target the RNA polymerases of HIV and other eukaryotic viruses select for escape mutations that increase viral replication rates [57], [58]. Growing arboviruses only in an arthropod cell line, rather than switching between their arthropod and vertebrate hosts, selects for substitutions that increase the production of the reverse transcriptase needed for genome replication [59]. Multiple studies on eukaryotic viruses indicate that growth in novel hosts leads to evolution in the viral-encoded polymerases to improve their interactions with host proteins such as RNA helicase and importin α [60], [61]. Another example of evolution of a foreign replication protein that interacts with a host helicase is found in bacterial host switching experiments with plasmids [62], [63]. These experiments lead to evolution of the plasmid replication initiation protein, especially in the region that interacts with the host helicase, leading to increased plasmid copy number, which is analogous to evolution in the replication initiation protein of φX174. Although molecular mechanisms by which evolution increases replication rates may be very different, especially in eukaryotic viruses, the process of genome replication is a major target for adaptation.
